# Pan-cancer analysis of integrin alpha family and prognosis validation in head and neck squamous cell carcinoma

**DOI:** 10.3389/fonc.2025.1556226

**Published:** 2026-01-12

**Authors:** Shaojie Ji, Fang Hao, Chenghui Zhang, Chunyan Hu, Henglei Ren, Qiang Huang, Jifeng Gu

**Affiliations:** 1 Department of Otorhinolaryngology, The Fifth Affiliated Hospital of Zhengzhou University, Zhengzhou, China; 2 Department of Pathology, Eye & ENT Hospital, Fudan University, Shanghai, China; 3 Department of Otorhinolaryngology, Eye & ENT Hospital, Fudan University, Shanghai, China; 4 Department of Pharmacy, Eye & ENT Hospital, Fudan University, Shanghai, China

**Keywords:** integrin alpha family, head and neck squamous cell carcinoma, prognosis, ITGA3, ITGA5, ITGA6

## Abstract

**Background:**

Integrins are cell-surface receptors involved in the interaction of cells with the extracellular matrix and are essential for processes such as cell adhesion, migration, and proliferation. However, the specific mechanisms by which integrin α family (ITGA) genes contribute to tumorigenesis and progression remain to be thoroughly explored.

**Methods:**

In this study, based on The Cancer Genome Atlas, we explored the differential expression of ITGA family genes in 33 types of tumors and normal tissues. Univariate COX regression was employed to analyze its association with the survival outcomes of pan-cancer patients. Online tools were utilized to analyze genetic changes of the genes, study the relationship with immune subtypes and evaluate immune cells, and analyze the relationship with tumor mutational burden and stemness. Additionally, immunohistochemistry experiments were conducted on Head and neck squamous cell carcinoma (HNSC) tissue samples to assess the impact of ITGA3 and others on patient prognosis.

**Results:**

ITGA family genes are abnormally expressed in most tumors and are significantly associated with poor prognosis. Genetic changes in these genes are mainly amplifications, and the mutation group has a poor prognosis. The differential expression of ITGA family genes is related to increased immune-related scores and immune cells. In HNSC, the high expression of ITGA3/5/6 serves as a biomarker for poor prognosis.

**Conclusion:**

Our research results indicate that ITGA family genes can serve as valuable prognostic biomarkers and therapeutic targets for tumors.

## Introduction

Head and neck squamous cell carcinoma (HNSC) poses a significant health concern. HNSC is a type of cancer that originates in the squamous cells lining the mucosal surfaces of the head and neck region, including areas such as the oral cavity, pharynx, larynx, and nasal cavity (1). The etiology of HNSC is multifactorial. Risk factors comprise tobacco use (both smoking and smokeless varieties), heavy alcohol consumption, human papillomavirus (HPV) infection (particularly in oropharyngeal cancers), and exposure to certain environmental carcinogens (2, 3). Clinically, HNSC can present with a diverse range of symptoms, including a persistent sore throat, hoarseness, difficulty swallowing, a lump or mass in the neck, and unexplained bleeding in the oral cavity or nose. The prognosis of HNSC depends on several factors, including the stage of the cancer at diagnosis, the location of the tumor, and the patient’s overall health. Early-stage cancers are generally more amenable to treatment and have a better prognosis. In contrast, advanced-stage HNSC can be more challenging to manage and tends to have a poorer prognosis.

Treatment options for HNSC typically involve a combination of surgery, radiation therapy, and chemotherapy (4, 5). In some cases, targeted therapy and immunotherapy may also be considered. The choice of treatment depends on the specific characteristics of the tumor and the patient’s overall health condition. Ongoing research in the field of HNSC focuses on understanding the molecular mechanisms underlying cancer development, identifying new biomarkers for early detection and prognosis, and developing more effective treatment strategies. This includes exploring the roles of genetic and epigenetic changes, immune responses, and the tumor microenvironment in HNSC progression (6). Identifying novel highly specific and sensitive biomarkers as well as new molecular targets is crucial for elucidating the molecular mechanism of HNSC and enhancing patient outcomes.

The integrin family is a transmembrane receptor protein family widely present on the cell surface (7). It is primarily responsible for recognizing specific components in the extracellular matrix, such as collagen, fibronectin, and laminin, and mediating the adhesion between cells and the extracellular matrix through binding to these components. Integrins belong to the heterodimeric surface receptor family and contain non-covalently linked α (ITGA) and β (ITGB) components (8). In the physiological processes of the human body, integrins play a crucial role. They participate in signal transduction by binding to the extracellular matrix (ECM) through the production of adhesins. Each integrin possesses a distinct activation cycle and exerts its effects through the cascaded amplification of multiple pathways, acting as a signal transducer across cell membranes. This function is crucial for cell migration and positioning during embryonic development. For instance, during the migration of neural crest cells, specific integrins interact with extracellular matrix components and guide cells to specific locations, thereby forming different tissues and organs (9). On the other hand, integrins participate in multiple stages such as the recruitment of inflammatory cells, the migration and proliferation of fibroblasts, and the regeneration of epithelial cells during wound healing.

Integrins are not only mechanical adhesion molecules but also act as signal receptors to transmit extracellular signals into cells and activate a series of intracellular signal pathways (10). For example, integrins can activate signal pathways such as mitogen-activated protein kinase (MAPK) and phosphatidylinositol 3-kinase (PI3K), regulating biological processes such as cell proliferation, differentiation, survival, and apoptosis (11, 12). In normal tissues, integrins maintain the structural and functional stability of tissues by regulating interactions between cells and the extracellular matrix. For example, in vascular endothelial cells, integrins bind to basement membrane components to maintain the integrity and permeability of blood vessels (13). In pathological processes, the integrin family is also of paramount importance. Particularly in tumors, integrins influence various cancer-related functions in the human body and are involved in processes such as the adhesion and metastasis of tumor cells. As cell surface receptors, integrins bind to various components of the ECM, such as collagen, fibronectin, and laminin, thereby mediating adhesion between tumor cells and the ECM. This adhesion provides physical support for tumor cells, enabling them to exist stably within the tumor microenvironment. For example, in breast cancer, the αvβ3 integrin on the surface of tumor cells binds to fibronectin in the ECM, enhancing the adhesion of tumor cells and promoting tumor growth and metastasis (14). Although preclinical studies have demonstrated the anti-tumor potential of integrin inhibition, clinical trials targeting specific integrins such as αVβ3/αVβ5 with agents like cilengitide failed to achieve significant clinical benefits (15). These disappointing outcomes may stem from several factors, including the narrow focus on a limited subset of integrins while neglecting the potential roles of others in tumor progression, the suboptimal pharmacokinetic properties and unintended signaling activation associated with conventional RGD-based inhibitors, as well as the inherent biological challenges posed by integrin functional redundancy, compensatory mechanisms, and tumor heterogeneity that collectively undermine the efficacy of single-target approaches.

In recent years, significant progress has been made in cancer treatment with the development of novel therapeutic methods and strategies. Immunotherapy, including immune checkpoint inhibitors (such as anti-PD-1/PD-L1 antibodies) and adoptive cell immunotherapy (such as CAR-T cell therapy), has emerged as a powerful approach to enhance the immune system’s ability to recognize and eliminate cancer cells (16, 17). Targeted therapies, which are designed to specifically inhibit oncogenic mutations or overexpressed proteins, have also shown promising results. For example, drugs targeting epidermal growth factor receptor (EGFR) mutations or BRAF mutations have been successfully used in the treatment of certain cancers (18). Moreover, combination therapies, such as the combination of immunotherapy and targeted therapy, or the combination of different immunotherapeutic agents, have demonstrated enhanced efficacy compared to single-agent treatments. Understanding the molecular mechanisms underlying these new treatment modalities is crucial for optimizing cancer treatment strategies. The ITGA family genes, which are implicated in tumorigenesis and progression, may potentially interact with these novel treatment strategies. For instance, ITGA family genes could serve as biomarkers to predict responses to immunotherapy or targeted therapy (19, 20). Additionally, they might represent novel therapeutic targets that could be combined with existing treatment modalities to improve patient outcomes. Future studies are warranted to explore these potential connections and develop more effective treatment regimens.

In summary, integrins promote the occurrence, development, and metastasis of tumors through multiple mechanisms, including enhancing the interaction between tumor cells and the ECM, regulating immune cell function, and participating in tumor angiogenesis within the tumor microenvironment. Therefore, integrins have emerged as a significant target for tumor treatment. Integrins, acting as cell adhesion molecules, are implicated in various cancers, including acute myeloid leukemia, glioma, and ovarian cancer (21–23). However, the specific mechanism and application value of the ITGA gene still need to be further explored. While previous studies have investigated the integrin family in tumors, this study presents a comprehensive pan-cancer analysis of the ITGA family genes, covering 33 types of tumors, which has not been reported before. Our study systematically explores the expression patterns, prognostic significance, genetic alterations, and relationships with the tumor microenvironment and other tumor characteristics (such as TMB, MSI, and tumor stemness). In particular, for head and neck squamous cell carcinoma (HNSC), we not only identified the potential value of ITGA3/5/6 through bioinformatics analysis but also validated their role in patient prognosis with IHC, providing new insights into the mechanism of ITGA family genes in HNSC. Moreover, by integrating multiple bioinformatics tools and databases, we established a relatively complete analytical framework, which could serve as a methodological reference for future related studies.

This study analyzed the expression profiles of 11 ITGA family genes, including ITGA1, ITGA2, ITGA2B, ITGA3, ITGA4, ITGA5, ITGA6, ITGA7, ITGA8, ITGA9, and ITGA10. First, the Cancer Genome Atlas (TCGA) was used to examine the differential expression of ITGA family genes in 33 tumor and normal tissues. Univariate COX regression analysis was performed to investigate the correlation between ITGA family genes and survival outcomes, including overall survival (OS), disease-specific survival (DSS), disease-free survival (DFS), and progression-free survival (PFS), across various cancers. The online tool cbioportal.org was employed to analyze genetic alterations in the 11 ITGA family genes across various cancers. To further investigate the relationship between ITGA family gene expression and immune subtypes across different tumor types, a multi-dimensional systematic evaluation of various immune cells was conducted using TIMER 2.0 (http://timer.comp-genomics.org). Additionally, the relationship between ITGA family gene expression and tumor mutational burden (TMB) as well as tumor stemness was analyzed. Based on the aforementioned analysis, immunohistochemical (IHC) experiments were conducted on 79 collected HNSC tissue samples to assess the differences in expression levels of ITGA3, ITGA5, and ITGA6. HNSC patients were stratified using the median value as the cut-off to compare survival differences between high and low expression groups. The findings of this study offer preliminary insights into the mechanisms of action of different ITGA genes across various cancers and may hold potential value for the clinical treatment of HNSC.

## Methods

### Data source

TCGA is a highly influential cancer research database (24). It encompasses 33 different types of cancers and has collected extensive multi-omics data of numerous cancer patients, including whole-genome sequencing, transcriptome sequencing, epigenetics data, and proteomics data. Through large-scale genomic analysis, TCGA aims to comprehensively reveal the molecular characteristics and pathogenesis of cancer. The TCGA database provides abundant resources for cancer research. Researchers can utilize these data for cancer molecular typing, searching for potential therapeutic targets, and predicting patient prognosis (25).

We downloaded the expression matrix and clinical information of 33 tumor genes from the TCGA (https://portal.gdc.cancer.gov/) database and selected 11 ITGA family genes for analysis (including ITGA1, ITGA2, ITGA2B, ITGA3, ITGA4, ITGA5, ITGA6, ITGA7, ITGA8, ITGA9, and ITGA10).

### Expression and clinical correlation analyses of ITGAs in pan-cancer

The expression and survival data from 33 cancers were integrated for differential expression analysis. Univariate COX regression was subsequently performed to investigate the relationship between ITGA family genes and survival outcomes, including OS, DSS, DFS, and PFS, across various cancers. Further analysis was conducted to examine how the average expression levels of ITGA genes varied across different stages of the disease. Statistical significance was determined at a significance level of P < 0.05.

### Correlation of ITGAs expression with gene mutations

The cBioPortal for Cancer Genomics (https://www.cbioportal.org/) is a crucial platform for cancer genomics data analysis and visualization (26). It integrates a large amount of multi-omics data from various cancer research projects, including information such as gene mutations, copy number variations, gene expression, and protein expression. It offers researchers a convenient tool for in-depth study of the molecular characteristics and pathogenesis of cancer (27). cBioPortal is of great significance for cancer research. It promotes data sharing and cooperation among different research teams and accelerates the progress of cancer research. The dataset used in this study is from the pan-cancer analysis of the ICGC/TCGA whole genome consortium. The gene changes of 11 ITGA family genes in pan-cancer were analyzed.

### Correlation between ITGAs expression and TME

We explored the relationship between ITGA family gene expression and immune subtypes in different tumor types. By performing immunological and stromal scoring for each tumor sample and conducting relevant analyses, we determined the correlation between ITGA family genes and immune/stromal scores (28). Through TIMER 2.0 (http://timer.comp-genomics.org) (29), we conducted a multi-dimensional systematic evaluation of different immune cells to analyze whether there is a relationship between ITGA family genes and different immune cells.

### Relationship between ITGAs expression and TMB/MSI

TMB refers to the total number of somatic mutations per million bases in the tumor genome. A higher TMB generally indicates that tumor cells have more gene mutations and may generate more neoantigens, thus being more easily recognized by the immune system (30). Tumor patients with high TMB may have a better response to immune checkpoint inhibitors (such as PD-1/PD-L1 inhibitors) (31). Therefore, TMB can also serve as a biomarker for predicting the prognosis and treatment effect of tumor patients.

MSI refers to the phenomenon where the length of microsatellite sequences changes due to functional defects in the DNA mismatch repair (MMR) system (32). According to the degree of instability, it can be divided into microsatellite high instability (MSI-High, MSI-H), microsatellite low instability (MSI-Low, MSI-L), and microsatellite stability (Microsatellite Stability, MSS). Tumors with MSI-H usually have higher immunogenicity, and patients may have a better response to immunotherapy (33). Therefore, MSI detection can also be used for tumor diagnosis, prognosis evaluation, and treatment decision-making.

We downloaded pan-cancer TMB, MSI, and stem cell score based on DNA methylation (DNAss: DNA methylation), RNA-based stem cell score (including RNAss: RNA expression) data, and explored the relationship between the expression of ITGA family genes and the above tumor characteristics in pan-cancer.

### Functional enrichment analysis of ITGA family genes

GeneMANIA (https://genemania.org/) integrates information from multiple data sources, including gene expression data GEO, protein-protein interaction data BioGRID, IntAct, genetic interaction data, to predict the function of genes and study gene regulatory networks during disease development (34). We obtained the protein interactions network of ITGA family genes through the GeneMANIA web site, and next, we performed functional enrichment analysis of ITGA family genes to explore the biological functions of their gene families and the potential regulatory mechanisms involved.

### Comprehensive bioinformatics analysis of ITGA3, ITGA5, and ITGA6 in HNSC

Further exploration was conducted on the expression differences of ITGA3, ITGA5, and ITGA6 between HNSC and paired tissues. The relationship between the expression data of ITGA3, ITGA5, and ITGA6 and the staging of HNSC was obtained through the UALCAN database (35). Subsequently, the prognostic value of ITGA3, ITGA5, and ITGA6 for HNSC patients was evaluated using ROC curves and nomogram graphics. Additionally, we performed single-gene analysis on ITGA3, ITGA5, and ITGA6 in HNSC by employing the Gene Set Enrichment Analysis (GSEA) (36).

### IHC staining and prognostic analysis

IHC staining analysis was performed on 79 postoperative paraffin section specimens of tumor tissues and control normal tissues pathologically confirmed as HNSC after surgical treatment from November 2017 to March 2022. HNSC tissue samples were obtained from the Department of Otorhinolaryngology, Eye & ENT Hospital, Fudan University. All participants provided written informed consent. The protocols were authorized by the Clinical Research Ethics Committee of the Eye & ENT Hospital, Fudan University, and the research was performed according to the stipulation of the Helsinki Declaration. All experiments were performed in accordance with relevant guidelines and regulations.

The antibody used for IHC staining is from Proteintech company (ITGA3: 66070-1; ITGA 5: 27224-1; ITGA6: 27189-1). The paraffin sections were stained by WELLBio company and panoramic scanned at 400 times magnification using a slide scanning device. Then, the analysis was conducted using Visiopharm software (37). The scanned IHC staining results were analyzed using the Deep Learning (AI) module of the Visiopharm software. Initially, all cell nuclei were identified, and the total cell count was calculated. The HDAB-DAB filter was applied to segment the regions of interest based on staining intensity thresholds, with corresponding Labels assigned to categorize nuclei as follows: negative (0), weakly positive (1+), moderately positive (2+), and strongly positive (3+). The number of nuclei per Label category was quantified. Subsequently, the Phenotype analysis module was employed to train and define distinct tissue compartments based on single or combined fluorescence channels. These compartments were segmented according to color-based zoning, and the number of positive cells within each designated compartment was calculated. Relevant clinical information of patients was collected to determine whether there are significant differences in the impact of different expression intensities of ITGA3, ITGA5, and ITGA6 on patient prognosis.

### Statistical analysis

The pan-cancer data related to ITGA family gene expression was extracted and analyzed by Perl programming language (version 5.30.0) and R language (version 4.12). The differential analysis of target genes was constructed by ggpubr, plyr, and ggsci packages. The survival, forestplot, and survminer packages were used for the survival analysis of target genes. The stemness analysis of ITGA family gene expression in pan-cancer was visualized through fmsb, reshape2, and RColorBrewer. The limma, org.Hs.eg.db, clusterProfiler, and enrichplot packages were applied for the functional enrichment analysis of target genes. All the above analysis results were considered statistically significant when P < 0.05. P < 0.05, < 0.01, and < 0.001 respectively mean “*”, “**”, and “***”.

## Results

### ITGAs expression in pan-cancer

**Figure 1:**
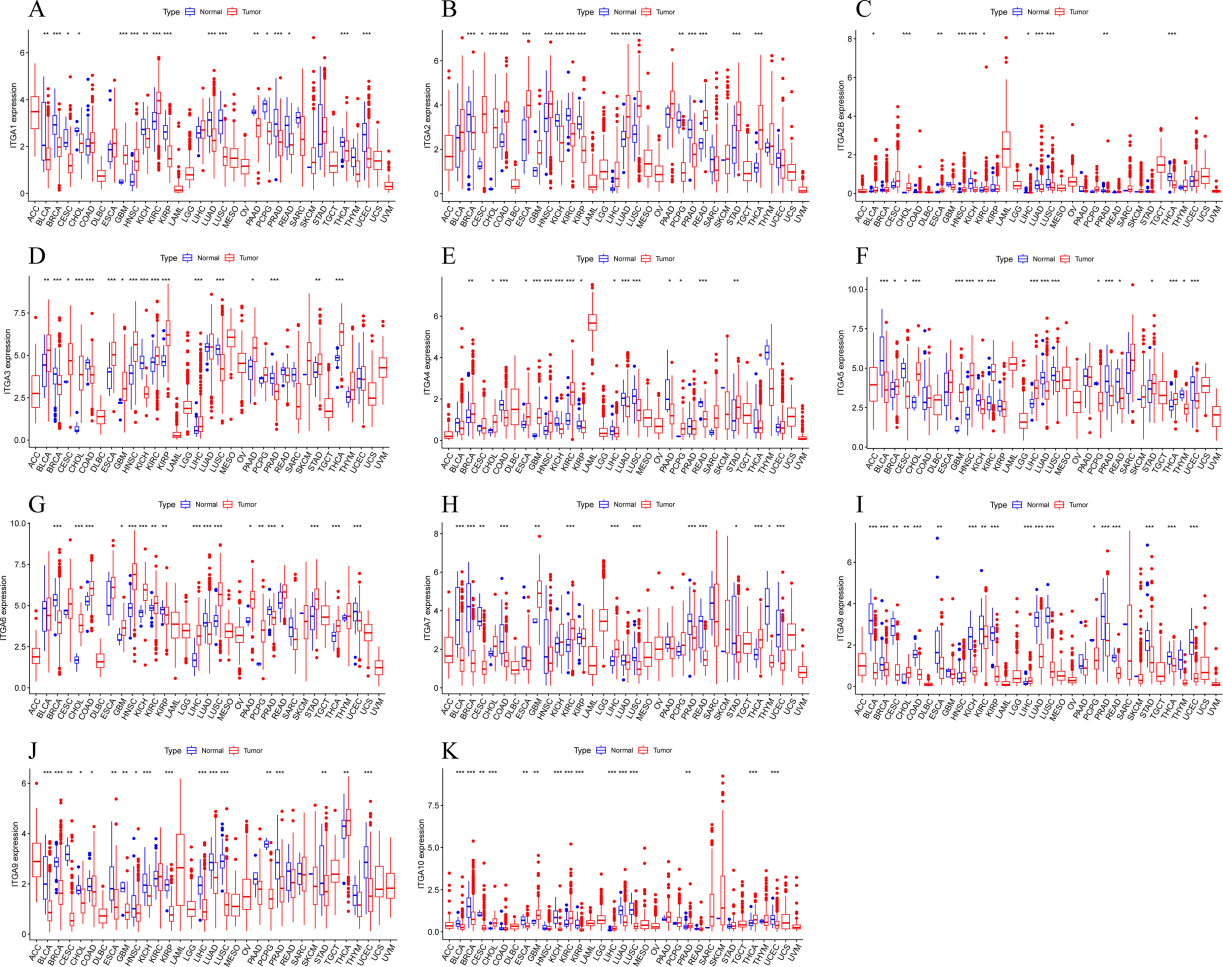
Differential expression of ITGA family in pan-cancer **(A)** ITGA1, **(B)** ITGA2, **(C)** ITGA2B, **(D)** ITGA3, **(E)** ITGA4, **(F)** ITGA5, **(G)** ITGA6, **(H)** ITGA7, **(I)** ITGA8, **(J)** ITGA9, **(K)** ITGA10.

The expression of ITGA family genes in TCGA normal tissues and tumor tissues was observed. It was found that the expression of ITGA family genes was abnormal in most tumor tissues. The expression level of ITGA1 was significantly decreased in tumor tissues such as bladder urothelial carcinoma (BLCA), breast invasive carcinoma (BRCA), kidney chromophobe (KICH), lung adenocarcinoma (LUAD), lung squamous cell carcinoma (LUSC), pancreatic adenocarcinoma (PAAD), prostate adenocarcinoma (PRAD), thyroid carcinoma (THCA), and uterine corpus endometrial carcinoma (UCEC), while the opposite was true in tumor tissues such as glioblastoma multiforme (GBM), HNSC, and kidney renal clear cell carcinoma (KIRC). Similarly, the analysis of the differential expression levels of other ITGA family genes is shown in **Figure 1**, and it was found that the expression levels of most ITGA family genes were significantly abnormal in tumor tissues. The expression level of ITGA7 was decreased in tumor tissues such as BLCA, BRCA, cervical squamous cell carcinoma and endocervical adenocarcinoma (CESC), colon adenocarcinoma (COAD), LUSC, PRAD, and UCEC.

### Prognostic analysis and tumor staging correlation analysis

**Figure 2:**
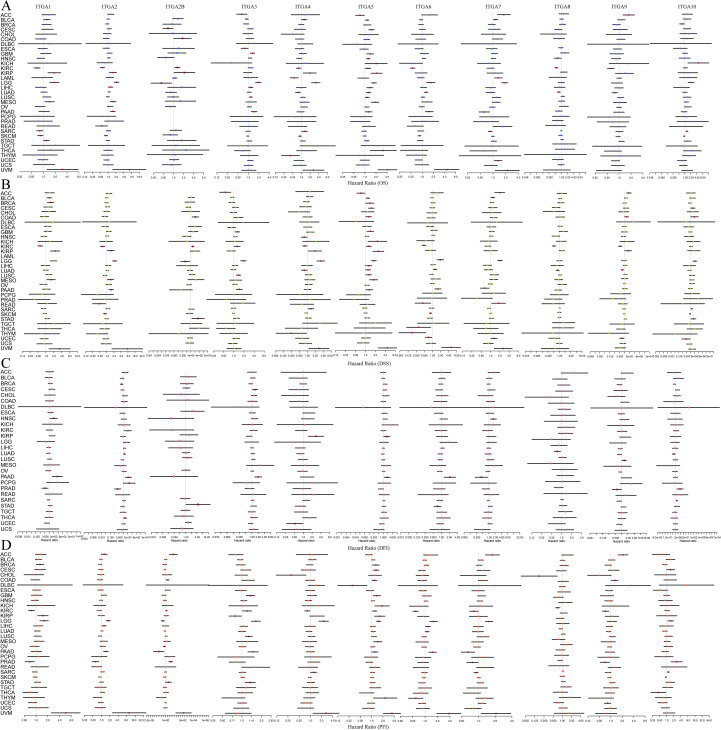
Univariate Cox regression analysis showing the association between ITGA family gene expression and survival in patients with various cancers **(A)** OS, **(B)** DSS, **(C)** DFI, **(D)** PFI.

Single-factor Cox regression analysis is a statistical method widely employed in medical research to assess the correlation between variables and their impact on survival outcomes. In this study, through single-factor Cox regression analysis, it was found that there is a significant association between the expression of ITGA family genes and OS, DFS, DSS, and PFS in various cancer types. As shown in **Figure 2A**, when conducting a comprehensive analysis of multiple cancers, a significant correlation is exhibited between OS and the expression of ITGA family genes. When the same method is applied to evaluate DFS, DSS, and PFS, comparable prognostic results are obtained. As shown in **Figures 2B-D**, these results further emphasize the important role of ITGA family gene expression in the survival prognosis of cancer patients.

**Figure 3:**
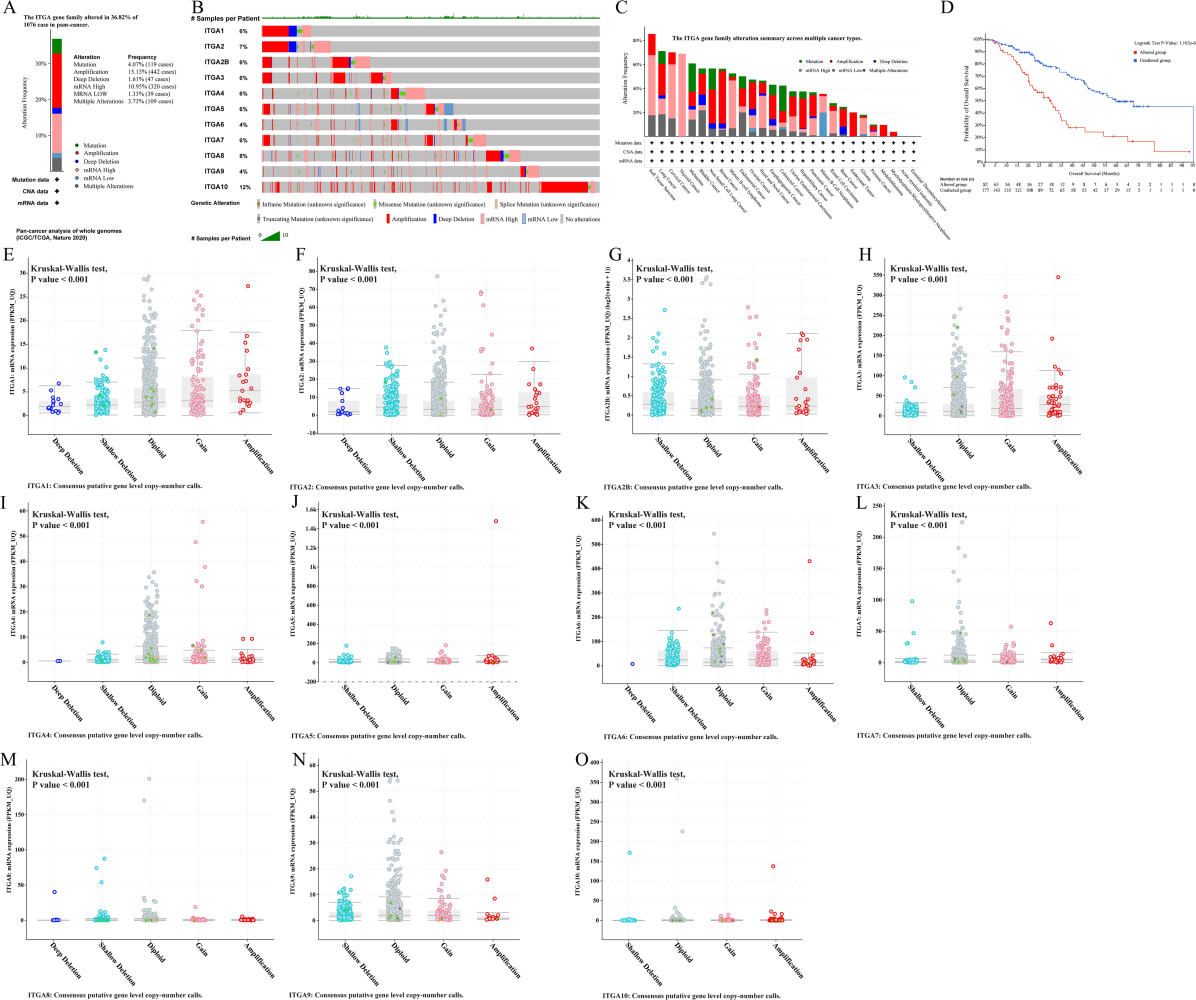
Genetic alterations of ITGA family genes **(A)** Frequency of ITGA family gene alterations in different cancers. **(B)** Type and extent of mutations in the ITGA family gene. **(C)** Summary of the types of mutations observed in the ITGA family gene. **(D)** Kaplan-Meier survival curves comparing overall survival in patients with and without ITGA gene alterations. copy number variations (CNVs) of ITGA family genes. analysis of CNVs of ITGA genes and their correlation with gene expression levels. **(E)** ITGA1, **(F)** ITGA2, **(G)** ITGA2B, **(H)** ITGA3, **(I)** ITGA4, **(J)** ITGA5, **(K)** ITGA6, **(L)** ITGA7, **(M)** ITGA8, **(N)** ITGA9 and **(O)** ITGA10.

Further in-depth analysis of the role of ITGA family gene expression levels in tumor progression suggests that tumor progression is closely related to the expression of ITGA family genes (**Figure 3**). Combined with the previous differential expression analysis, it is suggested that ITGA 3, ITGA 5, and ITGA 6 may contribute to the genesis and development of HNSC.

### Mutation analysis

**Figure 4:**
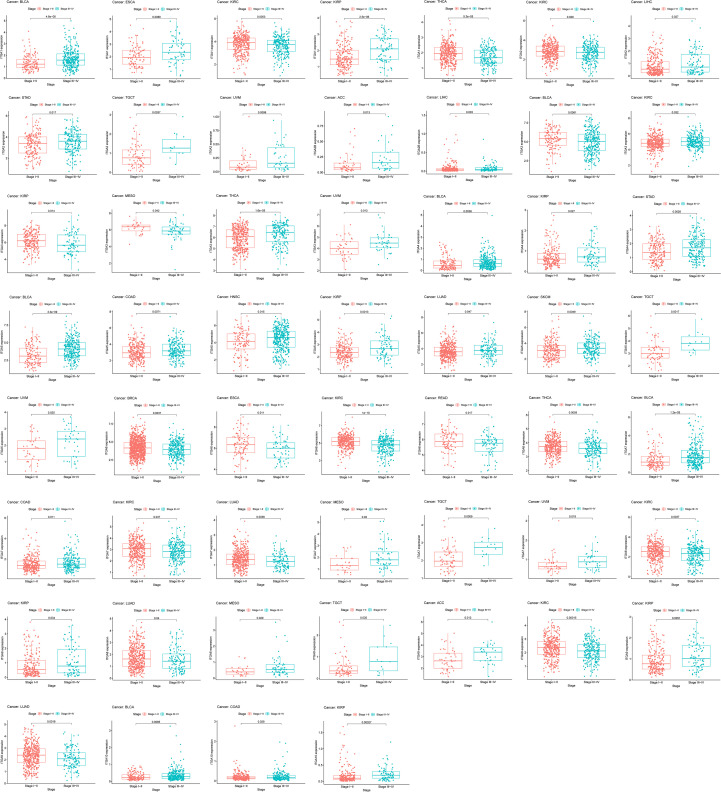
Relationship between ITGA family gene expression and STAGE grading of various tumors.

Analysis of targeted sequencing data reveals a relatively high frequency of genetic alterations in ITGA family genes, with amplification being the predominant form. It is found that 36.82% of cases exhibit alterations in ITGA family genes (**Figure 4A**). Specifically, the ITGA1 gene is altered in 6% of cases, and the ITGA10 gene is altered in 12% of cases (**Figure 4B**). As shown in **Figure 4C**, amplification is the main form of genomic alterations in the ITGA family and is detectable in multiple cancer types including LUSC, UCEC, ovarian cancer (OV), and BLCA. Patients with ITGA family genes in pan-cancer cases may experience reduced treatment efficacy when stratified based on these alterations. Moreover, compared to patients without ITGA family gene alterations, their median OS is significantly reduced (P < 0.001) (**Figure 4D**). As can be seen from the research results in **Figures 4E**–**O**, pan-cancer patients have a higher incidence of amplification and gain. Additionally, it is evident that as the copy number of CNV increases, the expression of most ITGA family genes shows an upward trend.

### TME and immune infiltration analysis

**Figure 5:**
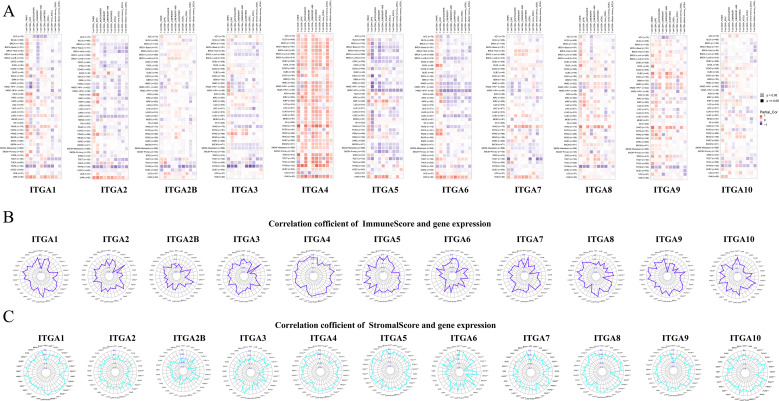
Relationship between ITGA family gene expression and immune-related indicators **(A)** Correlation between ITGA family gene expression and immune cell infiltration. ITGA family gene and ImmuneScore **(B)**. Correlation of ITGA family genes with StromalScore **(C)**.

The TME is the internal environment for tumor cell survival, proliferation, and metastasis. It consists of tumor cells themselves, various non-tumor cell components, and the extracellular matrix (ECM). Immune cells are an important constituent of the TME and participate in the formation of a tumor immunosuppressive microenvironment, which is one of the important mechanisms by which tumor cells evade attacks from the immune system (38). As shown in **Figure 5A**, we used a spearman correlation heatmap to study the correlation between immune cell infiltration in tumors and the expression of ITGA family genes. By analyzing the immune-related score of each tumor sample, we found a significant correlation between the differentially expressed ITGA family genes and the immune-related score. The radar chart further shows that in most of the 33 cancers, there is a positive correlation between the expression level of ITGA family genes and ImmuneScore and StromalScore (**Figures 5B, C**). This means that an increase in the expression of ITGA family genes is associated with an increase in immune cells in the patient’s tumor.

### TMB, MSI and tumor stemness analysis

**Figure 6:**
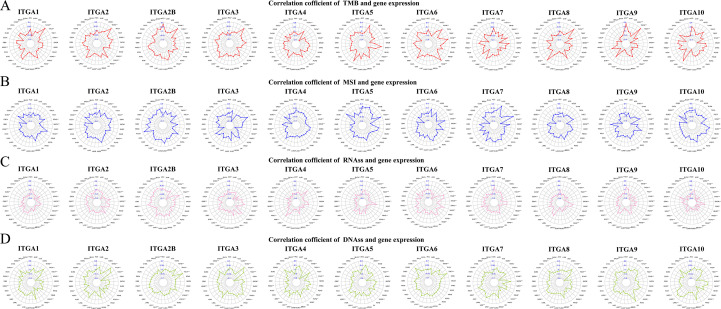
Association of ITGA family genes with TMB **(A)**. Relationship of ITGA family genes with MSI **(B)**. Relationship of ITGA family genes with RNAss **(C)**. Relationship of ITGA family genes with DNAss **(D)**.

We evaluated the correlation of each ITGA family gene and found that the expression of most ITGA family genes is significantly correlated with TMB (**Figure 6A**). Regarding microsatellite instability in pan-cancer, each ITGA family gene has a different correlation with MSI in different types of cancers (**Figure 6B**). Finally, in this study, RNAss and DNAss techniques were used to investigate the correlation between ITGA family genes and tumor stemness indicators in different types of cancers (**Figures 6C, D**).

### Functional enrichment analysis of ITGA family genes

**Figure 7:**
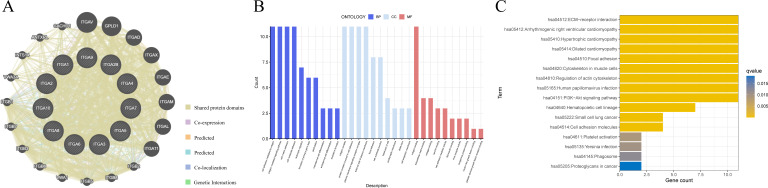
Functional enrichment analysis **(A)** Protein-protein interaction network of ITGA family genes. **(B)** GO analysis of ITGA family genes. **(C)** KEGG analysis of ITGA family genes.

Initially, we obtained the PPI network of ITGA family genes (**Figure 7A**), and the results suggested that there were shared protein regions among ITGA family genes, implying that possibly ITGA family genes play synergistic roles in the regulation of organismal physiopathology. The GO analysis indicated that the ITGA family genes were closely associated with cell-to-cell adhesion and interaction function with the ECM (**Figure 7B**). And KEGG analysis revealed that ITGA family genes were associated with ECM-receptor interactions and PI3K-Akt signaling pathway (**Figure 7C**).

### Comprehensive bioinformatics analysis of ITGA3, ITGA5, and ITGA6 in HNSC

**Figure 8:**
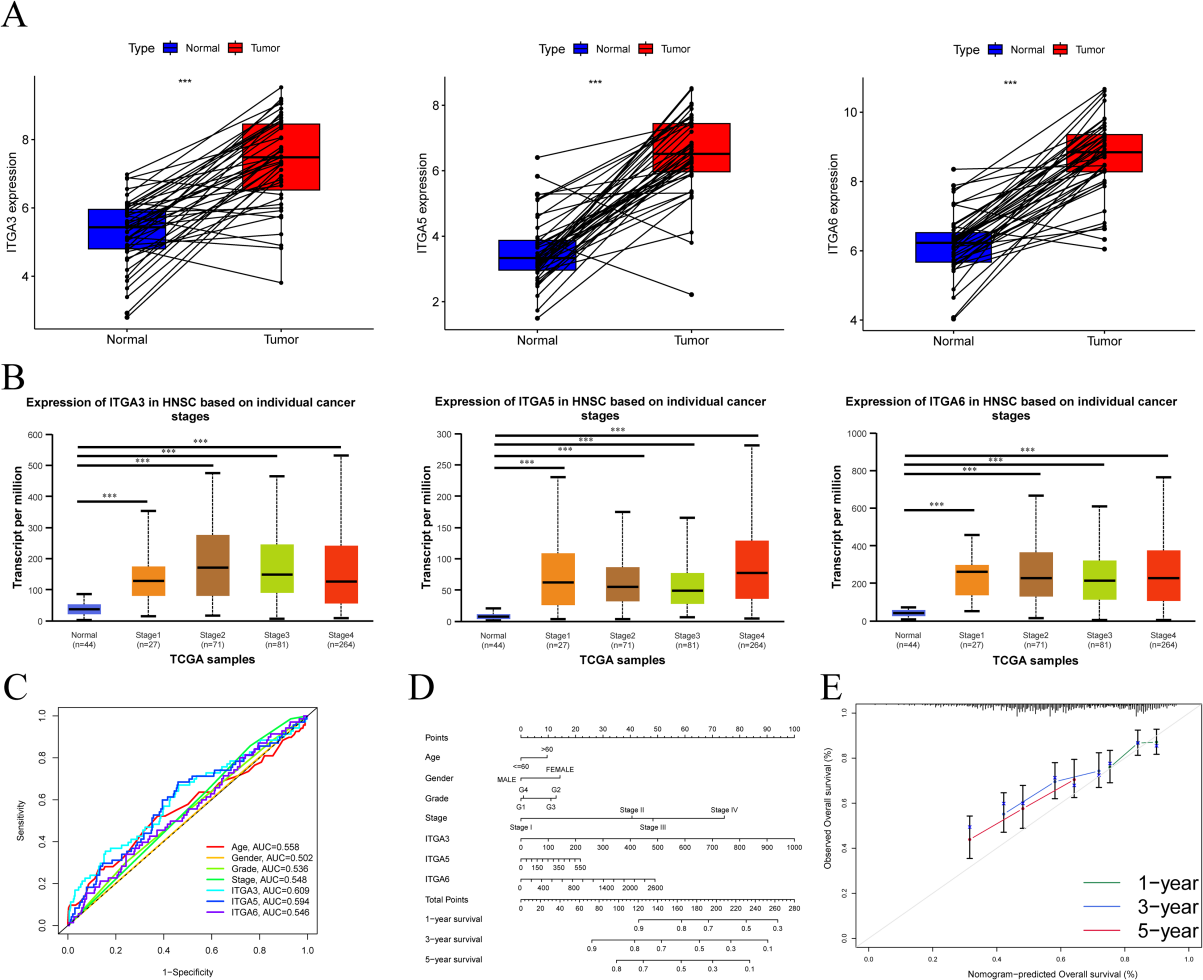
Analysis of the role of ITGA3/5/6 in HNSC **(A)** Differential expression of ITGA3/5/6in tumor tissues and paired tissues in HNSC. **(B)** Relationship between the staging of ITGA3/5/6 in cancer and gene expression in HNSC obtained by UALCAN database. **(C)** ROC curves of ITGA3/5/6 with HNSC. **(D)** nomogram curves and corrected curves **(E)** Evaluation of the prognostic value of ITGA3/5/6 in HNSC.

Further analysis of the expression relationship between tumor tissues and adjacent paired tissues of ITGA3, ITGA5, and ITGA6 in HNSC (**Figure 8A**) reveals that the expressions of ITGA3, ITGA5, and ITGA6 are significantly increased in tumor tissues. The UALCAN database suggests that the expression levels of ITGA3, ITGA5, and ITGA6 are related to the progression of HNSC (**Figure 8B**). Incorporating various clinical indicators (age, gender, grade, and stage) of HNSC and ITGA3, ITGA5, and ITGA6 together to draw the ROC curve, we find that the area under the AUC curve of ITGA3 (AUC = 0.609) is much larger than some clinical factors (**Figure 8C**). The nomogram curve and calibration curve suggest that ITGA3, ITGA5, and ITGA6 have excellent clinical predictive value in HNSC (**Figures 8D, E**).

### GSEA analysis in HNSC

**Figure 9:**
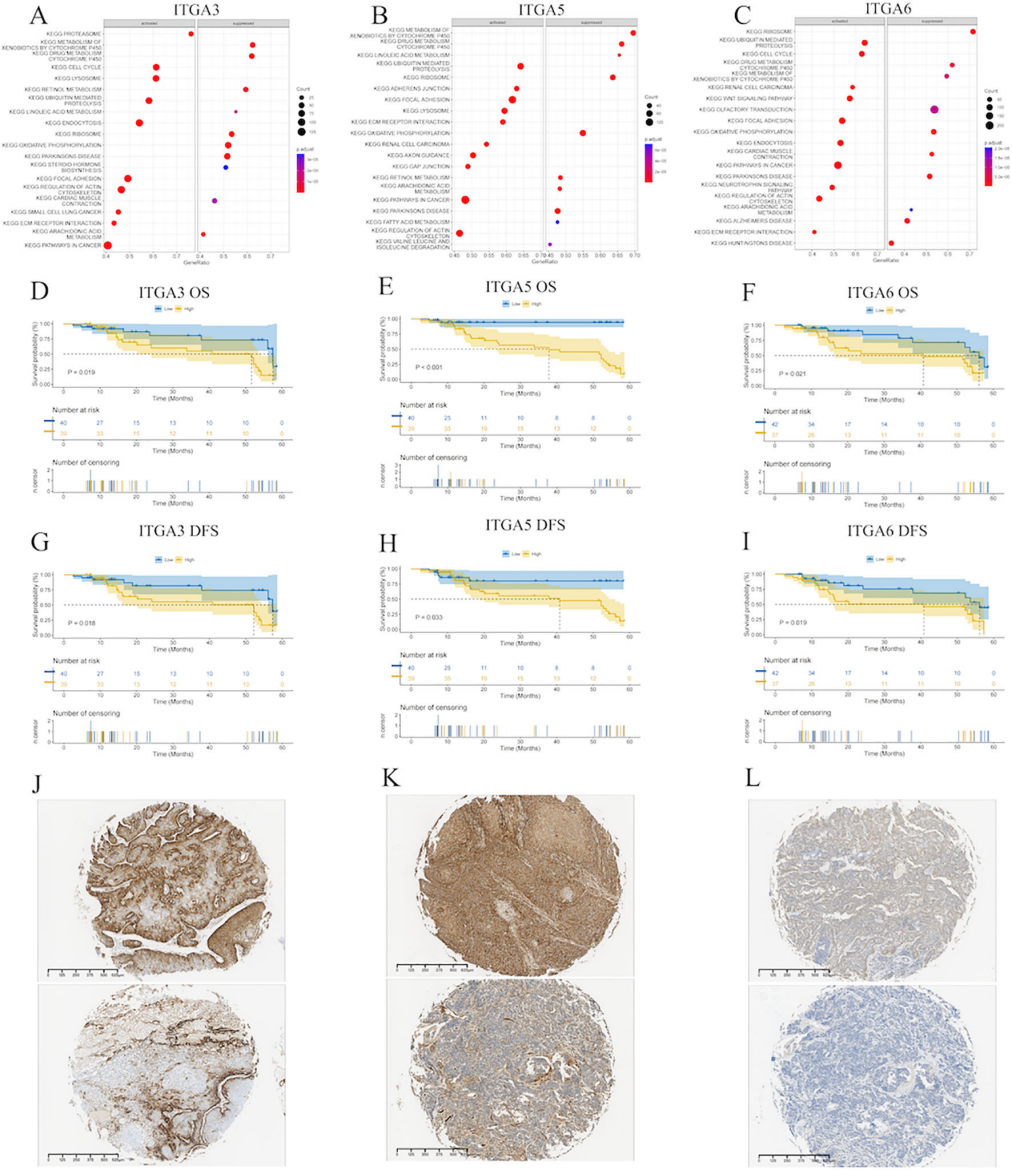
Single-gene GSEA analysis and survival curves of ITGA3/5/6 in HNSC **(A)** ITGA3, **(B)** ITGA5, **(C)** ITGA6 enriched pathways in HNSC. **(D)** OS of ITGA3, **(E)** OS of ITGA5, **(F)** OS of ITGA6, **(G)** DFS of ITGA3, **(H)** DFS of ITGA5, **(I)** DFS of ITGA6. Typical IHC images of ITGA3 **(J)**, ITGA5 **(K)**, and ITGA6 **(L)**.

Finally, we performed the functional enrichment analysis of ITGA3, ITGA5, and ITGA6 expression in HNSC using the GSEA method (**Figures 9A**–**C**). Analysis indicates that ITGA3, ITGA5, and ITGA6 have activating or inhibiting effects on multiple tumor-related pathways. Among them, ITGA3 and ITGA6 activate the KEGG cell cycle pathway, which may lead to abnormal cell proliferation. Normal regulation of the cell cycle is crucial for cell growth and division, and abnormal activation may cause uncontrolled cell proliferation, which is an important characteristic of tumorigenesis and development. In addition, ITGA3, ITGA5, and ITGA6 show activating effects on the KEGG pathway in cancer, which is consistent with previous analysis results (39). The cancer-related pathway covers multiple processes related to tumorigenesis and development, such as cell proliferation, apoptosis, angiogenesis, invasion, and metastasis. The activation of these genes may promote tumor progression by affecting multiple links.

### Clinicopathological features in HNSC

Tissue sections from patients and relevant follow-up information were collected. HNSC patients were divided using the median values of ITGA3, ITGA5, and ITGA6 as cut-off values. Their clinical characteristics are shown in **Table 1**. Survival curve analysis suggests (**Figures 9D**–**I**) that in both OS and DFS, increased expression of ITGA3, ITGA5, and ITGA6 often predicts a poor prognosis for patients. Typical immunohistochemical images of different expression levels of ITGA3, ITGA5, and ITGA6 are presented in **Figures 9J**–**L**, with the high expression group on top and the low expression group on the bottom.

**Table 1:** Relationship between the expression of ITGA3/5/6 and clinical information of HNSC.

	ITGA3				ITGA5				ITGA6				ITGA7			
Factors	Low N = 40	High N = 39	total	P	Low N = 40	High N = 39	total	P	Low N = 37	High N = 42	total	P	Low N = 40	High N = 39	total	P
Age																
<=60	15	12	27	0.528	17	10	27	0.114	14	13	27	0.520	12	15	27	0.428
>60	25	27	52		23	29	52		23	29	52		28	24	52	
Stage																
II	6	4	10	0.814	3	7	10	0.333	5	5	10	0.607	5	5	10	0.557
III	14	14	28		14	14	28		11	17	28		12	16	28	
IV	20	21	41		23	18	41		21	20	41		23	18	41	
Volume																
0-2	7	3	10	0.408	6	4	10	0.743	5	5	10	0.366	3	7	10	0.076
2-5	30	32	62		30	32	62		27	35	62		31	31	62	
>=5	3	4	7		4	3	7		5	2	7		6	1	7	
Number of lymph node metastases																
0	15	13	28	0.300	14	14	28	0.041	13	15	28	0.109	15	13	28	0.241
1-3	7	14	21		13	8	21		14	7	21		7	14	21	
>3	9	6	15		10	5	15		6	9	15		8	7	15	
NA	9	6	15		3	12	15		4	11	15		10	5	15	
Differentiation																
Low	1	0	1	0.534	0	1	1	0.029	1	0	1	<0.001	0	1	1	0.481
Moderate	28	30	58		25	33	58		16	42	58		31	27	58	
High	11	9	20		15	5	20		20	0	20		9	11	20	

## Discussion

Integrins are a family of transmembrane receptor proteins that are extensively present on the cell surface and serve an important role in tumorigenesis, progression as well as metastasis (8). Integrins promote the survival, proliferation, and differentiation of cancer cells by enhancing interactions with growth factors such as vascular endothelial growth factor receptors and epidermal growth factor receptors. Integrins can enhance the proliferation and survival of tumor cells by activating signaling pathways such as focal adhesion kinase (FAK) and PI3K (40, 41). Additionally, integrins can activate signaling pathways such as extracellular signal-regulated kinase (ERK) and c-Jun N-terminal kinase (JNK) to regulate the migration and invasion of tumor cells (42). The αvβ3 integrin can bind to vascular endothelial growth factor (VEGF), activate signal pathways such as PI3K/Akt and ERK, and promote the proliferation and migration of vascular endothelial cells, thereby promoting tumor angiogenesis (43). At the same time, integrins can also regulate the activation state of immune cells and affect their anti-tumor immune function. For example, the αvβ3 integrin can inhibit the activation of T cells and promote tumor immune escape (44). ​Integrins represent promising therapeutic targets in cancer treatment. Current strategies to directly inhibit integrin function include monoclonal antibodies, RGD-based peptide inhibitors, and allosteric modulators (45). Additionally, the combination of integrin inhibitors with anti-angiogenic agents has emerged as a key research focus. However, clinical trials targeting specific integrins such as αVβ3/αVβ5 with cilengitide failed to demonstrate significant improvements in patient outcomes (15). These challenges underscore the need for further mechanistic studies to develop more effective integrin-targeted therapies.

In this study, the potential roles and associated mechanisms of ITGA family genes in tumors were analyzed using the TCGA public database and HNSC tissue samples collected by our center. As the ITGA family has attracted our attention, its expression levels vary significantly across various tumors and are closely associated with important survival indicators such as OS, DSS, and PFS. It is widely acknowledged that there is an inseparable relationship between tumor occurrence and progression and gene mutations. Gene mutations are among the key driving factors for tumor formation. Common types of gene mutations include point mutations, insertions and deletions, gene amplification, and chromosomal translocations (46). Further analysis of the mutational landscape of ITGA family genes revealed that their genetic alteration frequency is relatively high, with amplification being the predominant form. Mutations in ITGA family genes were observed in 36.82% of cases, resulting in a poorer prognosis compared to the unmutated group. The mutation frequency of ITGA10 is relatively high, with changes observed in 12% of cases. ITGA10 is believed to drive osteosarcoma proliferation and chemotherapy resistance by mediating the PI3K/AKT pathway and is also considered a potential diagnostic and prognostic marker in THCA (47, 48).

TMB, as an indicator that can comprehensively assess the total number of somatic mutations occurring in tumor tissues, can serve as a biomarker for predicting tumor patients’ responses to immunotherapy (31). Tumors with high TMB typically possess a greater number of tumor neoantigens that can be recognized by the immune system as non-self-antigens, thereby activating T cell-mediated antitumor immune responses. Consequently, patients with high TMB may exhibit a more favorable response to immunotherapies, including immune checkpoint inhibitors. Radar chart analysis indicates that the expression levels of ITGA3 and ITGA7 are significantly negatively correlated with the TMB level in HNSC, suggesting that patients with higher expression levels of ITGA3 and ITGA7 have reduced immunotherapy efficacy. Analysis of clinical sample data further provided compelling evidence, indicating that patients in the ITGA3 high-expression group had poorer prognosis. Mismatch repair (MMR) is a crucial DNA repair mechanism within cells. Its primary function is to recognize and correct impaired bases generated during DNA replication, thereby maintaining genomic stability (32). When MMR is dysfunctional, it leads to MSI. Detection of MSI can help doctors select appropriate treatment plans and improve the treatment efficacy and survival rate of patients. ITGA5 and ITGA6 has been observed to be significantly negatively correlated with the MSI level of HNSC, suggesting that when the expression levels of ITGA5 and ITGA6 are higher, the degree of MSI instability is lower, and the sensitivity to immunotherapy is poorer, which is consistent with previous analysis results. Although the correlation between ITGA6 and TMB was modest, its strong association with immune cell infiltration suggests alternative mechanisms influencing HNSC progression. Survival analysis of clinical samples further substantiates that patients in the high ITGA5 and ITGA6 expression groups exhibit poorer prognosis.

The TME is the fundamental environment on which tumor tissues depend for survival. It comprises tumor cells themselves, immune cells, stromal cells, extracellular matrix, as well as various cytokines and signaling molecules (49). The TME plays a crucial role in the occurrence, development, and metastasis of tumors. An inhibitory TME can promote the growth and survival of tumor cells, provide an environment for tumor cells to escape immune surveillance, and facilitate tumor angiogenesis and invasion and metastasis (49). The role of infiltrating immune cells in the tumor microenvironment is complex and diverse. On the one hand, some immune cells such as cytotoxic T lymphocytes (CTLs) and natural killer (NK) cells have antitumor effects and can recognize and kill tumor cells. On the other hand, some immune cells such as regulatory T cells (Tregs) and M2-type macrophages have immunosuppressive effects and promote tumor progression (50). ImmuneScore and StromalScore can reflect the composition and activity of immune cells and stromal cells in the tumor microenvironment, providing quantitative indicators for understanding the characteristics of the TME (51). In the majority of tumor types, a significant positive correlation is observed between ITGA4 and immune-related scores. ITGA4 is believed to play a predictive role in the development and prognosis of various tumors. As demonstrated by the research of Fang et al., ITGA4 can enhance the proliferation, migration, and invasion of gastric cancer cells by modulating the tumor immune microenvironment (52). In patients with HNSC, a negative correlation between the expression levels of ITGA5 and ITGA6 and cytotoxic CD8^+^ T cells is observed across several datasets. This suggests that elevated expression levels of ITGA5 and ITGA6 may result in decreased infiltration of inhibitory immune cells in the TME, thereby promoting tumor progression.

Further in-depth analysis of the roles of ITGA3/5/6 in HNSC reveals that ITGA3/5/6 is closely related to multiple pathways in tumor development. For HNSC, ITGA3/5/6 likely plays an important role by activating signal pathways such as FAK and PI3K. Research has found that after ITGA3 binds to extracellular matrix components, it can trigger the phosphorylation of FAK, thereby activating downstream signaling molecules and promoting the proliferation of tumor cells (53). In an experiment on HNSC cell lines, after inhibiting the expression of ITGA5, the proliferation ability of tumor cells significantly decreased, accompanied by a reduction in the activity of the PI3K signaling pathway (54). This indicates that ITGA5 may promote the survival of tumor cells by activating the PI3K signaling pathway. The role of ITGA6 in HNSC cannot be ignored. Studies have shown that high expression of ITGA6 is closely related to the invasiveness of HNSC tumors (55). Our findings demonstrate that ​​ITGA3, ITGA5, and ITGA6​​ play ​​critical roles​​ in HNSC, a mechanistic insight that has ​​not been fully elucidated​​ in prior studies. Specifically, we observed that these integrins ​​not only regulate tumor cell adhesion, migration, and invasion​​ but also ​​modulate key signaling pathways​​ (*e.g.*, FAK/PI3K/AKT and TGF-β cascades) that drive HNSC progression. Importantly, ​​high expression levels of ITGA3/5/6 correlate with advanced tumor stage, lymph node metastasis, and poor patient survival​​, suggesting their potential as ​​prognostic biomarkers​​ and ​​therapeutic targets​​. While previous research has primarily focused on ​​classical integrins​​ in other cancer types, our study provides ​​novel mechanistic evidence​​ for the ​​distinct and synergistic contributions​​ of ITGA3/5/6 in HNSC pathogenesis, filling a critical gap in the current understanding of integrin-mediated oncogenesis.

Previous studies have reported the functions of ITGA family genes in promoting tumor cell proliferation, migration, and invasion, as well as their interactions with the immune microenvironment (56, 57). However, our study further refines and extends these findings. For example, in contrast to some previous studies that focused on specific tumor types or individual ITGA genes, our pan-cancer analysis provides a broader perspective on the overall behavior of the ITGA family genes. By comparing our results with those of previous studies, we found that differences in experimental methods, sample sources, and study populations may contribute to the observed discrepancies. For instance, some previous studies used cell lines, while our study is based on patient-derived samples from TCGA, which may better reflect the *in vivo* situation. Our findings suggest that ITGA3/5/6 play crucial roles in HNSC, which was not fully elucidated in previous studies. This knowledge could guide future research efforts to develop targeted therapies against these genes in HNSC and potentially other cancers. Additionally, our comprehensive analysis of the relationships between ITGA family genes and various tumor characteristics, such as TMB and MSI, provides novel insights into the potential mechanisms underlying tumor development and immune evasion, which could open new avenues for personalized cancer treatment. This study has certain limitations, such as the lack of in-depth mechanistic experimental validation for the identified signaling pathways. Functional studies are required to elucidate the mechanisms of ITGA3/5/6 in HNSC, which will be the focus of our next research phase.

In conclusion, ITGA3/5/6 is closely related to multiple pathways in the development of HNSC. By activating signal pathways such as FAK and PI3K, it plays an important role in promoting the proliferation, survival, and migration of tumor cells. These research results provide important clues for in-depth understanding of the pathogenesis of HNSC and also offer potential targets for the development of new treatment strategies. This study has provided emerging research directions. We can further explore the potential of ITGA-targeted therapies in HNSC and the combination of ITGA family targeting with immune checkpoint inhibitors. The importance of the ITGA family as a predictive biomarker should be validated in clinical trials.

## Conclusion

Our research results indicate that ITGA family genes can serve as valuable prognostic biomarkers and therapeutic targets for tumors. Further studies are necessary to fully elucidate the mechanism of action of ITGA genes in cancer progression and treatment.

## Data Availability

The original contributions presented in the study are included in the article/supplementary material. Further inquiries can be directed to the corresponding authors.
